# Fatigue‐Inducing Protocols in Parkinson’s Disease: Implications for Gait Assessment and Rehabilitation: A Systematic Review

**DOI:** 10.1155/padi/8822220

**Published:** 2026-01-05

**Authors:** Mahdi Majlesi, Elaheh Azadian, Nader Farahpour, Rezvan Bakhtiarian, Hadi Nobari

**Affiliations:** ^1^ Department of Sport Biomechanics, Ha.C., Islamic Azad University, Hamedan, Iran, azad.ac.ir; ^2^ Department of Motor Behavior, Ha.C., Islamic Azad University, Hamedan, Iran, azad.ac.ir; ^3^ Department of Sport Biomechanics, Faculty of Sport Sciences, Bu-Ali Sina University, Hamedan, Iran, basu.ac.ir; ^4^ Department of Sport Biomechanics, Ha.C., Islamic Azad University, Hamedan, Iran, azad.ac.ir; ^5^ LFE Research Group, Department of Health and Human Performance, Faculty of Physical Activity and Sport Science (INEF), Universidad Politécnica de Madrid, Madrid, Spain, upm.es; ^6^ Department of Exercise Physiology, Faculty of Educational Sciences and Psychology, University of Mohaghegh Ardabili, Ardabil, 56199-11367, Iran, uma.ac.ir

**Keywords:** biomechanics, fatigue, gait analysis, mobility, Parkinson’s disease

## Abstract

**Background:**

Fatigue is a common and disabling nonmotor symptom of Parkinson’s disease (PD), which significantly impacts gait and overall mobility. In spite of its clinical significance, the biomechanical consequences of different fatigue induction protocols on gait performance in PD are not yet well understood.

**Objective:**

To systematically review fatigue induction protocols in gait studies of individuals with PD and to examine how different types of fatigue (local, general, and cognitive) and assessment methods influence gait outcomes.

**Methods:**

In accordance with PRISMA guidelines registered under PROSPERO (CRD420251038246), five databases were systematically searched from January 2004 to March 2025. Seven studies met the inclusion criteria and were reviewed and analyzed through descriptive synthesis.

**Results:**

Repeated sit‐to‐stand tasks were the most effective in inducing lower‐limb fatigue and produced consistent changes in gait, including reduced stride length, slower speed, and impaired turning. General aerobic or functional tasks had inconsistent effects, and no study directly tested cognitive fatigue on gait. Fatigue assessment methods varied widely, including force decline, perceived exertion, and fatigue scales. Gait outcome measures were also heterogeneous, limiting comparability.

**Conclusion:**

Targeted lower‐limb fatigue protocols are effective in revealing gait impairments in PD. There is a clear need for standardized fatigue induction procedures and gait evaluation methods to improve consistency and comparability across research. Clinically, assessing gait under fatigue conditions may uncover subtle mobility impairments and inform more personalized rehabilitation strategies.

## 1. Introduction

Fatigue is a common and disabling symptom in Parkinson’s disease (PD), affecting roughly one‐third to one‐half of the patients [1, 2]. Fatigue is characterized by a significant reduction in energy or an excessive sense of effort that is disproportionate to the activity performed, and is commonly classified into peripheral and central types [3, 4]. Peripheral fatigue refers to muscle weakness and reduced force output because of repeated muscular activity (influenced by tremor, bradykinesia, etc.), whereas central fatigue—which can have physical and mental subtypes—manifests as persistent exhaustion without peripheral motor failure [5–7]. The pathophysiology of PD‐related fatigue is complex, involving both central (e.g. striatal‐limbic and neurotransmitter dysfunction) and peripheral mechanisms [8]. Indeed, it is often considered a nonmotor symptom with a large central component, although peripheral deconditioning and neuromuscular changes also likely contribute [9–11].

Clinically, fatigue in PD is strongly linked to reduced quality of life and functional impairment [12–14]. Patients often report that fatigue severely restricts their mobility and ability to carry out daily tasks [15]. Recent work has shown that higher self‐reported fatigue correlates with slower gait, shorter step length, and poorer endurance in PD [16, 17]. For example, fatigue severity independently predicts shorter distance on the 6‐min walk test (6MWT) [13]. In spite of its prevalence and impact, fatigue is often overlooked in routine assessments, and its interplay with motor function remains poorly understood. In particular, the question of how fatigue influences gait biomechanics in PD is critical, as gait impairments (like short shuffling steps, reduced speed, freezing, and balance deficits) are a hallmark of PD and major contributors to falls. Before proceeding, it is important to distinguish between fatigue, the subjective sense of exhaustion commonly reported by patients, and “fatigability,” the objectively measurable decline in motor performance during sustained activity. This review focuses primarily on experimentally induced fatigability and its biomechanical consequences on gait in PD, following the conceptual framework proposed by Kluger et al. [4].

The purpose of this systematic review is to evaluate the evidence on fatigue induction protocols used in gait assessment of individuals with PD, and to determine which fatigue protocol is most effective in producing biomechanical changes in gait. We specifically address: (1) Which fatigue induction protocols have been used in biomechanical gait studies in PD; (2) Differences between fatigue types (local muscular vs. general, physical vs. cognitive, or combined) in their effects on gait; and (3) Methods for quantifying fatigue and resulting functional changes, and the extent of standardization across studies. By synthesizing current evidence, this review aims to guide future research and clinical practice in integrating fatigue assessments into gait evaluations for PD.

## 2. Methods

### 2.1. Protocol and Registration

This systematic review was conducted following the guidelines of the Preferred Reporting Items for Systematic Reviews and Meta‐Analyses (PRISMA) [18]. The protocol was prospectively registered in PROSPERO (CRD420251038246).

### 2.2. Search Strategy

A comprehensive literature search was conducted using PubMed/MEDLINE, Web of Science, Scopus, IEEE Xplore, and Google Scholar. The search covered studies published from 1 January 2004, and was last updated on 17 March 2025, which was the date of the final database search. The search strategy combined terms related to PD, fatigue protocols, and gait analysis. Example search terms included: “Parkinson’s Disease” OR “PD” OR “Parkinsonian Gait” AND “Fatigue Protocols” OR “Fatigue Induction” OR “Motor Fatigue” OR “Fatigue” AND “Gait Assessment” OR “Gait Analysis” OR “Walking”. Filters were applied to include only English‐language articles and studies involving human participants. Reference lists of all included papers and relevant reviews were manually screened to identify any additional eligible studies. No restrictions were applied regarding study design to maximize coverage of available evidence.

### 2.3. Eligibility Criteria

Studies were eligible for inclusion if they met the following criteria: (a) included adults aged 50–85 years with idiopathic PD diagnosed according to the UK Brain Bank or Movement Disorder Society (MDS) criteria [19], typically Hoehn and Yahr stages 1–3 [20]; (b) reported the medication state of participants (ON or OFF dopaminergic medication) during gait assessment, or controlled for it analytically [21]; (c) involved the application of an objective fatigue induction protocol (e.g., repeated sit‐to‐stand (STS) tasks, treadmill walking, resistance exercises, or cognitive dual‐task paradigms); (d) assessed gait performance using validated biomechanical methods (e.g., motion capture, inertial sensors, or force plates) both before and after fatigue induction or in fatigued versus control conditions; (e) reported quantitative biomechanical gait outcomes, including spatiotemporal parameters, kinematic or kinetic measures, and balance indices; and (f) were published in peer‐reviewed journals in English. Exclusion criteria were: (a) studies focusing solely on subjective fatigue without an objective fatigue induction protocol; (b) studies reporting nongait outcomes only (e.g., cardiovascular, electrophysiological, or psychological parameters); (c) nonoriginal publications such as reviews, editorials, or conference abstracts lacking full data; and (d) studies involving participants with cognitive impairment (MMSE < 27), musculoskeletal disorders, or use of β‐blockers affecting cardiovascular or fatigue responses [22].

### 2.4. Study Selection

**Figure 1:**
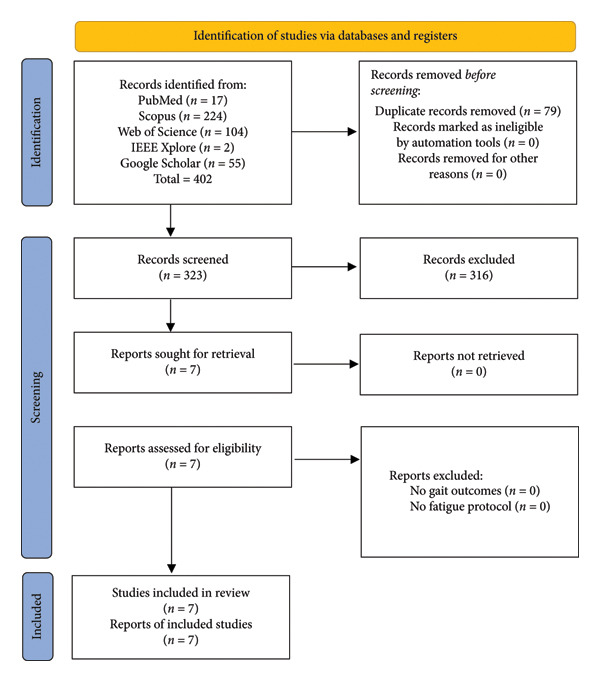
Prisma flow diagram.

Two independent reviewers screened all titles and abstracts according to the predefined inclusion and exclusion criteria. Subsequently, the full texts of potentially eligible articles were independently reviewed for final inclusion. Discrepancies between reviewers were resolved through discussion and consensus or by consultation with a third reviewer. Inter‐rater reliability between the two reviewers was assessed using Cohen’s kappa (*κ* = 0.83), indicating strong agreement. The overall selection process, including identification, screening, exclusion, and inclusion of studies, is summarized in Figure 1.

### 2.5. Data Extraction

Data were independently extracted by two reviewers using a standardized data extraction form, including:

• Study characteristics: first author, publication year, and study design;
• Participant details: sample size, demographic characteristics, and PD severity;
• Fatigue protocol details: type of fatigue (local muscle vs. general body, physical vs. cognitive), protocol duration, intensity, and methods of fatigue quantification;
• Gait assessment details: measurement instruments and methodologies (motion capture systems, force platforms, and inertial measurement units);
• Primary outcomes: quantitative changes in gait parameters (stride length, stride velocity, step width, cadence, double support time, and gait variability) because of fatigue;
• Secondary outcomes: balance measures, mobility tests (timed up and go test, turning assessments), and methods used to quantify fatigue levels (e.g., maximal force reduction, ratings of perceived exertion, and fatigue scales);
• Comparisons performed (pre‐ vs. post‐fatigue, healthy controls vs. PD patients, active vs. inactive PD patients).

To ensure accuracy, extracted data were cross‐checked between reviewers, and disagreements were resolved through consensus.

### 2.6. Quality Assessment

Risk of bias and methodological quality were independently evaluated by two reviewers. For randomized controlled trials, the PEDro scale was applied [23]; for nonrandomized studies, the ROBINS‐I tool was used [24]. Inter‐rater reliability for quality scoring was high (*κ* = 0.81). Any disagreements were resolved through consensus or by involving a third reviewer.

### 2.7. Data Synthesis

Given the methodological and clinical variability among the included studies, conducting a meta‐analysis was not appropriate Therefore, a narrative synthesis approach was adopted, structured around the review’s primary and secondary questions. Results were summarized descriptively in tables organized by the type of fatigue protocol and measurement outcomes. A PRISMA flow diagram illustrating the study selection process was included in the results. Common patterns and discrepancies in gait biomechanical changes induced by different fatigue protocols were identified and discussed. The certainty of evidence for primary outcomes was assessed using the grading of recommendations, assessment, development, and evaluations (GRADE) framework, considering the risk of bias, inconsistency, indirectness, imprecision, and publication bias [25].

## 3. Results

This section summarizes the findings from the seven included studies, integrating results across study characteristics, fatigue protocols, gait‐related outcomes, and methods used to quantify fatigue and functional change. The synthesis emphasizes analytical comparisons between fatigue types, gait parameters, and methodological variability.

### 3.1. Study Selection and Characteristics

The initial database search identified 402 records. After removing duplicates, 323 unique studies remained for screening. Of these, 316 were excluded for not meeting inclusion criteria (e.g., no PD sample, no gait outcomes, or no absence of fatigue protocols). Ultimately, seven studies published between 2016 and 2023 fulfilled all criteria and were included in the qualitative synthesis ([9, 17, 19–21, 26, 27]; Figure 1). Their main characteristics are summarized in Table 1, reflecting the growing research interest in fatigue‐related gait alterations in PD.

**Table 1:** Overview of included studies on fatigue protocols in PD gait assessment.

Study (Year)	Design & sample (PD = Parkinson’s disease; Ctrl = control)	Fatigue type & protocol	Gait assessment method	Key outcomes under fatigue
Bryant et al. (2016) [21]	Cross‐sectional; 45 PD (HY stage 2–3); treadmill test to max. Effort. No control group.	General physical fatigue – Graded treadmill exercise test (modified Bruce protocol) until exhaustion.	Cardiovascular measures: Exercise duration noted (no kinematic gait analysis).	Fatigue limited performance (primary reason for test stopping) with no falls. Cardiovascular responses (HR, BP) to maximal gait effort did not differ by PD severity. Safe to perform treadmill stress in PD.
Martino et al. (2016) [20]	Observational; 20 PD with fatigue (PD‐F) versus 20 PD without fatigue (PD‐NF); no controls.	Combined cognitive + motor fatigue – 5‐min finger tapping sequence task with external pacing (attention‐demanding) versus uncued task. Fatigue = performance decrement over time.	Not applicable to gait (outcome was finger tapping performance).	PD‐F showed greater motor performance worsening over 5 min in the attention‐demanding task (↑inter‐tap interval, ↓speed) versus PD‐NF. Subjective fatigue severity correlated with performance decline. No gait outcomes measured but suggests cognitive fatigue affects motor output.
Santos et al. (2016) [26]	Experimental pre‐post; 20 PD (mean HY ∼2.5) and 20 healthy controls; each subdivided into active versus inactive (physical activity level).	Local muscle fatigue—Repeated sit‐to‐stand (STS) from chair at self‐paced rapid rate until fatigue (Lower limb fatigue protocol, duration ∼1 min).	3D gait analysis on level walkway (pre‐ vs. post‐fatigue); measured stride length, velocity, duration, step width, % double support, plus ground reaction impulses.	After fatigue, both PD and controls increased stride length & velocity and reduced stride time (*p* < 0.05). Controls showed adaptive changes (↑step width, ↓double‐support) post‐fatigue, but PD did not change step width or double‐support. PD had wider steps and longer double support than controls at baseline. Physical activity level: Active versus inactive had baseline gait differences but did not significantly alter fatigue effects. Interpretation: Groups sought stability after fatigue; PD unable to adjust certain gait parameters.
Huang et al. (2017) [19]	Experimental pre‐post; 25 PD versus 25 healthy controls (age‐matched).	Local muscle fatigue—Isometric knee extension exercise (quadriceps) to fatigue. Measured MVC force loss to quantify peripheral fatigue, and central activation failure for central fatigue.	Gait speed was measured (10‐m walk) before and after fatigue; also assessed muscle strength pre/post and subjective fatigue scales (MFI, FSS).	PD had greater central fatigue (↓voluntary activation) and more subjective fatigue than controls. Peripheral fatigue (force loss) was similar between groups. Both groups had some reduction in walking speed after muscle fatigue; in PD, the drop in gait speed correlated with loss of peripheral strength, not with central fatigue indices. Concludes PD fatigue is largely central, but peripheral weakness impacts gait slowing.
Baer et al. (2018) [9]	Crossover experimental (within‐subject); 27 PD tested in ON‐med and OFF‐med states (1 week apart); no separate control group.	General functional fatigue—A sustained mobility task (fatiguing condition) interposed between balance/gait tests (likely fast walking or stepping exercise, ∼several minutes). Fatigue level not quantified in detail.	Comprehensive: Mini‐BESTest (balance score), instrumented walkway for spatiotemporal gait (preferred pace), and dynamic posturography tests, done pre‐ and post‐fatigue on each day.	No significant pre‐post differences in gait or balance outcomes because of the fatiguing task. Fatigue did not worsen gait characteristics or balance scores in PD in either medication state. Also, no interaction with medication (ON vs. OFF)—being on levodopa did not change fatigue’s effect. BDNF genotype had no effect on fatigue response. Concludes an acute fatiguing bout did not impair gait/balance in this PD sample.
Dos Santos et al. (2021) [27]	Experimental pre‐post: 10 PD‐active, 10 PD‐inactive, 10 control‐active, 10 control‐inactive (40 total).	Local muscle fatigue—Repeated STS (rSTS) to fatigue, like 2016 study. Maximal leg force measured pre/post to confirm fatigue.	Gait with obstacle negotiation: Subjects walked 8 m and stepped over a mid‐path obstacle (height ∼10% of leg length) before and after fatigue. Measured approach stride length, step width, speed, and crossing step parameters (lead/trail foot clearance).	Pre‐fatigue: Inactive PD had significantly shorter, slower strides and longer step duration approaching the obstacle versus others. Post‐fatigue: Inactive PD patients increased stride length and velocity by ∼23%–34% and decreased step width by 21% (*p* < 0.01)—effectively improving gait parameters. After fatigue, PD‐inactive approached the obstacle *similarly to controls*. Other groups had smaller changes. Being physically active buffered fatigue effects, maintaining more stable gait. Conclusion: Physical activity confers protection against fatigue‐related gait impairment, and fatigue may *reduce gait caution* in inactive PD.
Abd Ghani et al. (2023) [17]	Experimental pre‐post; 40 PD divided by severity (20 PD HY 2, 20 PD HY 3); no control group.	Local muscle fatigue—Sit‐to‐stand repetitions at 30 cycles/min from a standard chair until fatigued (both legs). Pre versus post comparison.	Extended timed up and go (TUG) test (10‐m walk with 180° turn) performed immediately before and after fatigue. Gait speed, stride length (via video analysis), and turning performance (steps, time) were recorded.	Fatigue significantly worsened gait: Stride length and gait velocity dropped post‐fatigue compared to pre (*p* = 0.001). Effects were more pronounced in HY 3 (moderate PD) than in HY 2. Both groups experienced greater turning difficulty when fatigued— needing > 5 steps and > 3s to complete a turn. Clinical implication: Lower limb fatigue can further impair gait and turning in PD, especially in more advanced stages, potentially increasing fall risk.

*Note:* Key outcomes are abbreviated from the original results for brevity.

Abbreviations: HY = Hoehn &Yahr stage; PD‐F = Parkinson’s with fatigue; MVC = maximum voluntary contraction; mini‐BESTest = Mini Balance Evaluation Systems Test.

Participants in all studies had idiopathic PD, generally with mild‐to‐moderate disease severity (Hoehn & Yahr stages 1–3). Sample sizes ranged from 20 to 45 participants, and three studies included healthy control groups [19, 26, 27]. Some further stratified subjects by physical activity level (active vs. inactive) [26, 27] or fatigue status (fatigued vs. nonfatigued) [20]. One study examined both ON‐ and OFF‐medication states [9]. The average participant age was in the 60s, with both sexes being represented. Chronic fatigue symptoms were prevalent, though definitions and scales varied. Most studies used within‐subject pre–post designs, assessing gait before and after a fatiguing intervention [17, 26]. Martino et al. (2016) employed a cross‐sectional comparison between fatigued and nonfatigued PD groups [20]. Baer et al. (2018) conducted a small interventional crossover study comparing medication states and exploring genetic modulation (BDNF polymorphism) of fatigue response [9]. Across designs, the consistent analytical framework was comparing gait performance in fatigued versus nonfatigued conditions, either experimentally induced or symptomatically classified.

### 3.2. Methodological Quality and Risk of Bias

Methodological quality and risk‐of‐bias ratings are summarized in Table 2. Three studies were rated as low risk and four as moderate risk; none were classified as high risk. Inter‐rater reliability between reviewers was substantial (*κ* = 0.81). The main methodological limitations were small sample sizes, lack of control groups, and incomplete blinding. Overall, the studies demonstrated adequate internal validity, supporting cautious synthesis of their findings.

**Table 2:** Summary of methodological quality and risk of bias in the included studies.

Study (author, year)	Study design	Assessment tool	Overall risk of bias	Main methodological limitations
Bryant et al. (2016) [21]	Observational	ROBINS‐I	Moderate	No control group; fatigue quantification limited
Martino et al. (2016) [20]	Observational	ROBINS‐I	Moderate	Nongait outcomes only; indirect inference to gait fatigue
Santos et al. (2016) [26]	Experimental	ROBINS‐I	Low	Small sample; short protocol duration
Huang et al. (2017) [19]	Experimental	ROBINS‐I	Low	Limited external validity; short post‐fatigue test
Baer et al. (2018) [9]	Crossover	PEDro	Moderate	Fatigue intensity not quantified; no blinding
Dos Santos et al. (2021) [27]	Experimental	ROBINS‐I	Low	Small sample; partial blinding
Abd Ghani et al. (2023) [17]	Experimental	ROBINS‐I	Moderate	No control group; subjective fatigue endpoint

### 3.3. Fatigue Induction Protocols

Fatigue protocols varied considerably in type, duration, and targeted mechanism (muscular vs. systemic vs. cognitive). Most studies focused on peripheral muscular fatigue of the lower limbs, whereas central or cognitive fatigue was rarely isolated. Repeated STS tasks were the most common paradigm [17, 26, 27], inducing localized quadriceps fatigue through 1–3 min of continuous, rapid chair rises (approximately 30 cycles·min^−1^) performed until exhaustion. Fatigue was verified either by a decline in maximal knee‐extension force [27] or by participant exhaustion. Isometric knee‐extensor protocols [19] isolated quadriceps fatigue, distinguishing peripheral from central fatigue through measures of twitch interpolation and voluntary activation. Graded treadmill tests [21] produced whole‐body fatigue via incremental workloads to volitional exhaustion, reflecting combined aerobic and muscular demand. Functional mobility tasks, such as short sustained walking or stepping sequences [9], elicited moderate global fatigue integrated within balance assessments. Only one study [20] examined cognitive‐attentional fatigability through a prolonged finger‐tapping task, indirectly addressing central fatigue mechanisms rather than gait performance. Across studies, fatigue intensity ranged from mild acute muscular exhaustion to maximal effort. No adverse events were reported. Verification methods for fatigue were heterogeneous: objective indices such as force loss [19, 27] and heart‐rate endpoints were the most robust, whereas others relied on subjective exhaustion [9, 26]. This heterogeneity underscores the absence of a unified fatigue‐induction standard in PD research.

### 3.4. Effects of Different Fatigue Types on Gait Performance

In spite of methodological diversity, convergent patterns emerged regarding how different fatigue types influence gait biomechanics in PD.

#### 3.4.1. Peripheral (Local Muscular) Fatigue

Lower‐limb fatigue generally resulted in reduced stride length, slower walking velocity, and prolonged turning time, reflecting a deterioration in dynamic stability. Abd Ghani et al. (2023) [17] reported significant post‐fatigue declines in gait velocity and stride length (*p* = 0.001) during the extended timed up and go test, particularly among individuals with Hoehn & Yahr stage 3 PD. Turning performance also worsened, with increases of more than 3 s in turning time and over five additional steps required to complete a 180° turn, confirming impaired mobility under fatigue. In contrast, Santos et al. (2016) [26] observed a transient improvement following fatigue—specifically, increased stride length and walking speed in both PD and control groups. They interpreted this as evidence of neuromuscular potentiation or a short‐term “warm‐up” compensatory effect. However, PD participants failed to adjust stability‐related parameters (step width and double‐support time) as controls did, suggesting impaired adaptive balance mechanisms. Similarly, Dos Santos et al. (2021) [27] found a physical activity–dependent effect: after STS‐induced fatigue, sedentary PD participants increased stride length and velocity by approximately 25%–30%, approaching values seen in controls, whereas active PD participants maintained stable gait performance. The authors interpreted this paradoxical improvement as a possible reduction in gait inhibition or short‐term facilitation following acute exertion.

#### 3.4.2. General (Whole‐Body) Fatigue

Bryant et al. (2016) [21] used a maximal treadmill protocol. Although gait kinematics were not directly measured, fatigue—not musculoskeletal instability—was the limiting factor, suggesting PD patients can safely reach exhaustion under supervision. No disease‐stage effect on endurance was noted, indicating generalized fatigability independent of severity. Similarly, Baer et al. (2018) [9] reported no measurable post‐fatigue decline in gait or balance parameters, possibly because of mild fatigue stimulus or effective compensatory control. These findings imply that PD patients can sustain moderate exertion without acute gait degradation when fatigue remains submaximal.

#### 3.4.3. Cognitive or Central Fatigue

No study directly tested mental‐fatigue effects on gait, a clear gap. However, Martino et al. (2016) [20] demonstrated that fatigued PD participants exhibited greater performance decline in an attention‐demanding tapping task than nonfatigued peers, implying vulnerability of central attentional mechanisms. Huang et al. (2017) [19] found that PD patients exhibited greater central fatigue (lower voluntary activation) than controls, but gait slowing correlated with peripheral rather than central indices, indicating a predominantly muscular contribution to immediate gait impairment.

#### 3.4.4. Comparative Synthesis and Heterogeneity

Collectively, these results reveal that fatigue effects on gait in PD are heterogeneous and task‐specific. Physical‐fatigue paradigms tend to worsen gait stability, while certain moderate‐intensity protocols may transiently enhance stride parameters through potentiation. The variability likely stems from differences in fatigue type (muscle vs. central), medication state, and physical conditioning. Qualitatively, effect magnitudes across studies corresponded to medium standardized differences (Cohen’s d ≈ 0.4–0.6) for changes in gait speed or stride length. This analytical comparison highlights compensatory versus maladaptive fatigue responses and underscores the need for standardized fatigue‐induction and gait‐analysis protocols.

### 3.5. Assessment of Fatigue and Functional Changes—Standardization of Methods

The seven studies used diverse methods to quantify fatigue and functional gait outcomes, reflecting the absence of a unified assessment framework.

The quantification of fatigue varied across studies. Chronic fatigue was typically measured using validated questionnaires such as the Parkinson fatigue scale (PFS‐16) or fatigue severity scale (FSS) [20]. Acute, task‐induced fatigue was verified through objective physiological indices—declines in maximal voluntary contraction (MVC) or voluntary activation [19, 27] —or through subjective exhaustion and heart‐rate endpoints [9, 21]. However, no study applied a standardized metric such as time‐to‐fatigue or a composite fatigability score, which impedes direct cross‐study comparison. Developing a unified fatigability index (e.g., %MVC loss or time to task failure) would help harmonize future PD fatigue research.

The assessment of gait and functional outcomes was consistent across studies, with all investigations quantifying spatiotemporal gait parameters—such as stride length, walking speed, cadence, step width, and double‐support time—using instrumented walkways or motion‐capture systems [26, 27]. Several studies incorporated additional functional tasks, including obstacle crossing [27] and turning performance within the extended timed up and go (TUG) test [17]. Balance‐related outcomes, such as the mini‐BESTest and sensory organization components, were included in one study [9]. Post‐fatigue gait measurements were obtained immediately (within 2 min), ensuring the capture of acute fatigue effects rather than recovery patterns. While gait speed and stride length were universally reported, the range of secondary outcomes varied substantially, limiting opportunities for meta‐analytic synthesis. Establishing a core outcome set—including at minimum gait speed, stride length, and double‐support percentage—would improve consistency and cross‐study comparability. Moreover, combining subjective fatigue scales with objective fatigability metrics could yield a more integrated understanding of fatigue–gait interactions. Among the existing protocols, the repeated STS task—applied in multiple studies [17, 26, 27]—emerges as a practical, reproducible, and clinically applicable fatigue‐induction paradigm that could serve as a foundation for a standardized “fatigue‐challenge” framework in PD gait assessment.

Overall, evidence from these seven studies demonstrates that fatigue, particularly peripheral muscular fatigue, significantly alters gait biomechanics in PD, manifesting as reduced stride length, velocity, and turning efficiency. However, under certain conditions, mild or moderate fatigue may transiently facilitate gait kinematics, possibly through neuromuscular potentiation or altered motor‐control strategies. These mixed responses underline substantial heterogeneity in fatigue mechanisms, protocol intensity, and participant conditioning, emphasizing the need for standardized methodological frameworks in future research.

## 4. Discussion

This systematic review synthesized recent findings on how various fatigue‐induction protocols influence gait biomechanics in PD. In spite of methodological heterogeneity, converging evidence indicates that fatigue—particularly peripheral muscular fatigue—modifies spatiotemporal gait parameters, turning performance, and balance control. Below, we clarify conceptual distinctions, interpret the divergent findings mechanistically, and highlight clinical implications for assessment and rehabilitation.

### 4.1. Conceptual Clarification: Fatigue, Fatigability, and Subjective Fatigue

Before interpreting the results, it is important to distinguish between fatigue—the subjective sense of tiredness—and fatigability—the objectively measurable decline in performance—following the taxonomy proposed by Kluger et al. (2013) and earlier conceptualizations by Lou (2009) [4, 15]. Subjective fatigue in PD often reflects central mechanisms such as dopaminergic depletion, limbic dysfunction, and reduced motivation, whereas fatigability reflects neuromuscular and metabolic limitations [28, 29]. Most studies included in this review examined motor fatigability, elicited through experimental physical tasks rather than chronic subjective fatigue. Dopaminergic state and freezing of gait (FoG) likely mediate fatigue‐related gait deterioration. During OFF‐medication phases, reduced striatal dopamine elevates perceived effort and decreases motor drive, potentially worsening gait and increasing freezing susceptibility [13, 29, 30]. Hence, future studies should clearly report medication state and account for FoG when interpreting fatigue–gait interactions.

### 4.2. Efficacy of Fatigue Protocols

Repeated STS exercises proved to be a practical and robust method for eliciting gait alterations associated with fatigue. Across multiple studies [17, 26], this localized muscle‐fatigue task consistently produced measurable changes in gait (velocity, stride length, and cadence). It targets the anti‐gravity muscles essential for locomotion, directly influencing walking ability when fatigued [31]. In contrast, general endurance or balance exercises often failed to produce comparable short‐term effects [9]. Thus, if the goal is to observe gait degradation because of fatigue, localized lower‐limb fatigue protocols (e.g., repeated chair stands or loaded step‐ups) appear more effective [32].

The treadmill maximal test [21] also induced global fatigue safely, though gait parameters were not reported. Huang et al. (2017) [19] employed an isolated quadriceps fatigue protocol allowing quantification of both central and peripheral components via voluntary activation and twitch‐interpolation techniques—highly precise but laboratory‐intensive. Overall, localized high‐intensity fatigue protocols such as STS remain the most effective for eliciting acute gait changes in PD.

### 4.3. Local versus Central (Physical vs. Cognitive) Fatigue Effects

Physical fatigue of the lower limbs exerts an immediate and observable impact on gait, whereas central or cognitive fatigue effects are less pronounced acutely. Several studies demonstrated slowed gait, shorter stride length, and impaired dynamic stability after peripheral fatigue [17, 26]. Conversely, central‐fatigue paradigms (e.g., prolonged finger tapping [20]) did not markedly affect gait in the short term. Peripheral fatigue arises from local energy depletion and reduced force generation, while central fatigue reflects decreased neural drive within cortical–subcortical circuits [33, 34]. Quadriceps and STS protocols predominantly elicit peripheral fatigue, whereas cognitive challenges engage higher‐order attentional mechanisms [9, 20]. Interestingly, certain studies reported paradoxical post‐fatigue improvements in stride length or speed [26, 27]. These transient enhancements may reflect neuromuscular potentiation or compensatory cortical activation [30, 31], consistent with neurophysiological evidence that transient facilitation can occur before decompensation. Over time, as central fatigue accumulates, compensatory mechanisms fail, resulting in slower gait and greater instability [35, 36]. Clinically, this biphasic pattern implies that mild exertion can transiently “normalize” gait (“warming‐up” effect), whereas sustained fatigue leads to deterioration. The absence of immediate gait decline post‐fatigue does not signify irrelevance; rather, different fatigue intensities or durations may be required to reveal deficits [28, 29, 35].

### 4.4. Standardization and Assessment

There remains a clear need for standardized protocols to quantify both fatigue and gait outcomes. Current studies employ heterogeneous metrics—subjective scales (PFS‐16, FSS) [28, 37], objective physiological measures (MVC reduction) [19, 27], and heart rate or RPE criteria [9, 21]. We recommend that future research: (a) verify fatigue induction using ≥ 15%–20% MVC reduction or RPE ≥ 17 and (b) report a core outcome set comprising gait speed, stride length, and double‐support percentage. The STS protocol (∼30 cycles min^−1^ for 1–3 min) is practical, safe, and reproducible [17, 26, 27]. Combining it with wearable‐sensor gait analysis could detect subtle fatigue‐related changes. Dos Santos et al. (2021) [27] showed that physically active PD participants exhibited less gait impairment after fatigue, implying enhanced fatigue resilience through habitual activity. Training programs emphasizing lower‐limb strength and endurance may raise fatigue thresholds and stabilize gait under exertion [38, 39]. Conversely, sedentary individuals experience gait deterioration even after mild fatigue, heightening fall risk [32, 40].

### 4.5. Clinical Implications

Including a fatigue component in gait assessment can reveal impairments not evident during brief, rested evaluations. For example, a patient may demonstrate normal walking over a 10‐m distance but exhibit significant gait slowing or instability after sustained exertion [41–43]. Simple standardized protocols—such as a 2‐min continuous walk or repeated STS task followed by gait analysis—can help clinicians identify fatigue‐induced gait deterioration [44, 45]. Turning performance appears particularly sensitive to fatigue [46]; therefore, clinicians are encouraged to assess turning and dual‐task walking after exertion to better predict fall risk in everyday situations. Rehabilitation strategies should concurrently target strength, endurance, and balance, as lower‐limb resistance and aerobic training have been shown to improve gait economy and delay fatigue onset [47–49]. In addition, multimodal and rhythm‐based interventions, such as dance‐based therapy (e.g., Argentine Tango), have demonstrated measurable benefits for balance, dynamic stability, and fatigue resilience through combined sensorimotor and cognitive engagement [50]. These programs likely facilitate cortical–subcortical synchronization and adaptive motor control, offering a holistic framework for fatigue management in PD rehabilitation.

Patient education remains essential: individuals with PD should be informed that fatigue amplifies gait instability and fall risk, especially later in the day or after prolonged activity. Energy‐conservation techniques, pacing strategies, and scheduled rest intervals should be incorporated into daily routines [51]. Although no falls were reported in the controlled experimental settings of the included studies [21], real‐world variability and cumulative fatigue effects increase fall risk, reinforcing the importance of integrating fatigue‐management principles within clinical gait evaluation and rehabilitation programs.

### 4.6. Limitations and Research Gaps

Only seven studies met the inclusion criteria, underscoring the scarcity of direct investigations into fatigue‐induced gait alterations in PD rather than reflecting an incomplete search process. The small sample sizes and diverse protocols among these studies limit generalizability and highlight the early developmental stage of this research area. Most available studies examined the acute effects of fatigue rather than its cumulative or long‐term manifestations. Future research should therefore implement cognitive or dual‐task fatigue paradigms—such as prolonged sustained‐attention or Stroop challenges preceding gait testing—to better simulate everyday cognitive exertion [20, 36]. Moreover, comparing gait and fatigue responses in ON versus OFF dopaminergic medication states is essential to isolate pharmacological influences [13, 29]. Continuous home‐based monitoring using wearable sensors could provide valuable insight into daily fatigue–gait fluctuations in real‐world conditions [52, 53]. In addition, controlled intervention trials examining pharmacologic or behavioral anti‐fatigue strategies could determine whether improving fatigue tolerance enhances gait endurance and stability. The interplay between central fatigue, motivation, and motor performance remains complex; some researchers suggest that fatigue in PD partially overlaps with apathy or bradykinesia [30]. Advanced neuroimaging and EMG‐based approaches may help disentangle these mechanisms. Ultimately, establishing unified fatigue‐challenge protocols and standardized outcome measures will facilitate meta‐analytic synthesis and accelerate clinical translation.

## 5. Conclusion

Current evidence indicates that short‐duration, high‐intensity lower‐limb fatigue protocols such as repeated STS maneuvers are effective in eliciting acute gait impairments in individuals with PD, characterized by reduced gait velocity, decreased stride length, and compromised turning performance. In contrast, fatigue induced through general aerobic or cognitive tasks demonstrates inconsistent effects on gait parameters and remains insufficiently investigated. The reviewed protocols exhibit considerable heterogeneity, with localized muscular fatigue being the most frequently employed and most directly associated with observable gait alterations. Nevertheless, the absence of standardized fatigue induction and gait assessment methodologies limits the comparability of findings across studies and impedes the development of unified clinical guidelines. From a clinical perspective, incorporating fatigue‐inducing tasks into gait evaluations may unmask mobility deficits that are not apparent under rested conditions, thereby enhancing the ecological validity of assessments. Rehabilitation strategies should address both fatigue resistance and gait adaptation to improve functional mobility under real‐world conditions. Future investigations should prioritize the development of standardized fatigue protocols, include paradigms that target cognitive fatigue, and evaluate long‐term functional outcomes to advance the understanding and management of fatigue‐related gait disturbances in PD.

## Disclosure

All authors approved the final version of the manuscript.

## Conflicts of Interest

The authors declare no conflicts of interest.

## Author Contributions

Study conception and design: all authors; first reviewer: MM; second reviewer: HN; draft manuscript preparation: EA. All authors provided feedback on all versions of the manuscript.

## Funding

No funding was received for this manuscript.

## Data Availability

The data that support the findings of this study are available from the corresponding author upon reasonable request.
